# Corrosion Resistance Analysis in Nickel Coatings by Electrodeposition with Different Layers and Waveform Combinations

**DOI:** 10.3390/ma18010002

**Published:** 2024-12-24

**Authors:** Liang Yan, Tao Zhang, Huajin Zhang, Huan Liu

**Affiliations:** 1 School of Electromechanical and Vehicle Engineering, Zhengzhou University of Technology, Zhengzhou 450044, China; 2 School of Mechanical and Power Engineering, Henan Polytechnic University, Jiaozuo 454003, China; zt17638938659@163.com (T.Z.); 19513576305@163.com (H.Z.); liuhuan@hpu.edu.cn (H.L.)

**Keywords:** electrodeposit, nickel coating, pulse waveforms, pulse combinations, corrosion resistance

## Abstract

Nickel (Ni) single-layer coatings were electrodeposited under varying pulse periods (T), duty cycles (θ), and average current densities (i_av_) using four distinct pulse current waveforms: rectangular (Rec), triangular (Tri), ramp-up triangular (Rup), and ramp-down triangular (Rdn). This study demonstrated, through dynamic polarization curves and surface morphology analysis, that single-layer coatings showed relatively good corrosion resistance when deposited at shorter pulse periods, larger duty cycles, and higher average current densities. Moreover, compared with other pulse current waveforms, single-layer coatings electrodeposited at T = 10 ms, θ = 0.5, and i_av_ = 10 mA/cm^2^, 20 mA/cm^2^, and 40 mA/cm^2^ with Rdn had similar dynamic polarization curves and relatively good corrosion resistance. Consequently, two pulse current combinations, the descending gradient and convex gradient, were introduced for electrodepositing Ni multilayer coatings. Analysis revealed that the corrosion resistance of coatings deposited with the convex gradient current was further enhanced.

## 1. Introduction

Nickel (Ni) exhibits excellent ductility, high-temperature strength, corrosion resistance, and fatigue resistance, making it widely utilized in various applications, including aerospace, marine turbines, medical devices, nuclear power systems, and the petrochemical industry [1]. The main preparation methods of Ni coating are physical vapor deposition, chemical vapor deposition, and electrodeposition [2,3,4]. Electrodeposited technology is widely used to prepare Ni coatings because of its low cost and simple equipment [5,6]. To enhance the corrosion resistance of Ni coatings in complex corrosive environments and to meet the requirements of various fields, recent research has focused on improving Ni coating performance and surface morphology by incorporating metal elements (e.g., Mo, Al, Co) or modifying the electrolyte composition with additives [7,8,9,10,11]. However, these methods increase processing costs and require more stringent processing conditions [12]. Pulse electrodeposition technology was introduced to optimize the process and reduce costs while maintaining the mechanical and chemical properties of Ni coatings [13]. Pulse electrodeposition is a technique to improve the material properties by periodically regulating the current or voltage. Research and production experience have demonstrated that applying pulse techniques significantly enhances the corrosion resistance, hardness, compactness, and resistivity of the Ni deposition layer, compared to conventional electrodeposited technology [14,15].

Currently, Ni coatings produced through electrodeposition technology have garnered significant attention, with rectangular waveform electrodeposition being the conventional method. Sajjadnejad et al. [16] electrodeposited nanocrystalline Ni coatings at different current densities, duty cycles, and pulse frequencies. The results showed that the passivation layer had better corrosion resistance in the (211) grain orientation than in the (100) grain orientation. Sivasakthi et al. [17] electrodeposited Ni coatings on low-carbon steel by pulse current in sulfate electrolytes and concluded that corrosion resistance and charge transfer resistance were enhanced at a low duty cycle and low frequency. Nasirpouri et al. [18] electrodeposited Ni coatings using ultrasonic-assisted direct current (DC) and pulse current (PC). The results showed that PC could reduce the grain size and effectively improve the corrosion resistance of the coatings. Tang et al. [19] electrodeposited Ni coatings with diminished grain size and decreased porosity in PC. After that, the corrosion resistance of Ni coatings was improved by introducing pulse reversal plating. Wen et al. [20] reported that organic additives and reverse-pulsed electrodeposition altered the grain size and texture orientation of Ni coatings. The addition of saccharin could reduce the grain size of nanocrystalline Ni coatings, and the corrosion resistance of the coatings became better with the decrease in crystallite size.

In addition to the above rectangular wave research, in the field of pulse electrodeposition of Ni coatings, Geramipour et al. [21] electrodeposited Ni–Cu alloy coatings on low carbon steel by changing the pulse current waveforms, including triangular, sinusoidal, ramp-up triangular, and ramp-down triangular currents. The dynamic polarization curve and electrochemical impedance spectroscopy (EIS) showed that coatings electrodeposited with triangular waveforms exhibited the highest corrosion resistance under identical electrical parameters. Moreover, compared to single-layer Ni coatings, multilayer coatings demonstrated a reduction in cracks, pores, and defects, attributed to the preferential nucleation and growth at high-energy defect sites in the underlying layer. Imaz et al. [22] electrodeposited double-layer Ni coatings using pulse electrodeposition, which exhibited transverse diffusion of corrosion and effectively slowed the corrosion of the substrate.

In summary, in the field of electrodeposition of Ni coatings, the influence of pulse waveforms on the electrodeposition and corrosion resistance of coatings, as well as the electrodeposition of single-layer and multilayer coatings, still has a lot of research space. In this paper, four distinct pulse current waveforms, rectangular (Rec), triangular (Tri), ramp-up triangular (Rup), and ramp-down triangular (Rdn), were employed to electrodeposit Ni coatings. The effects of different pulse periods (T), duty cycles (θ), and average current densities (i_av_) on the corrosion resistance of single-layer coatings were systematically analyzed. The parameters and pulse current waveforms with relatively good corrosion were selected, and methods for electrodepositing multilayer coatings under different current density combinations, including the current density gradient (Layer 1) and the convex gradient average current density (Layer 2), were proposed. Additionally, the corrosion resistance of the multilayer coatings was analyzed.

## 2. Materials and Methods

### 2.1. Experiment Setup and Process

**Figure 1:**
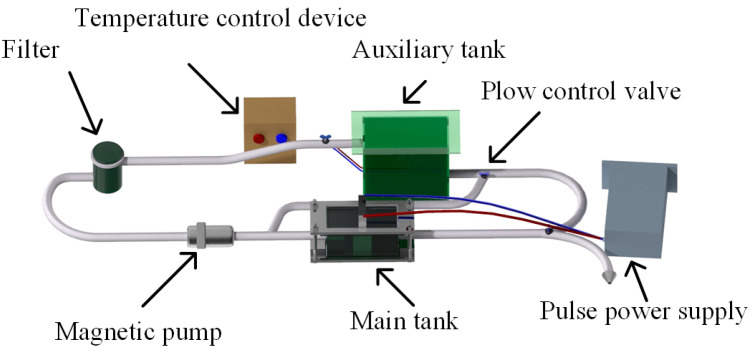
Electrodeposition Ni coatings equipment.

**Figure 2:**
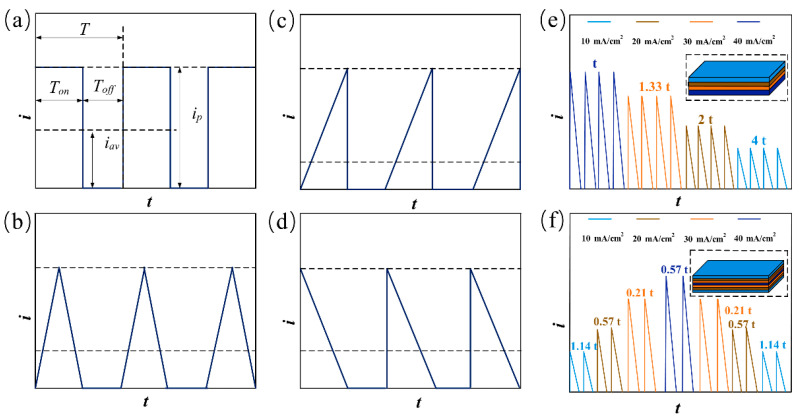
Pulse current waveforms and pulse combinations diagrams: (**a**) Rec; (**b**) Tri; (**c**) Rup; and (**d**) Rdn. (**e**) Layer 1; (**f**) Layer 2.

The electrodeposition Ni coatings equipment is shown in Figure 1. It consisted of a pulse power supply, a main tank, an auxiliary tank, a temperature control device, a filter, a magnetic pump, and several flow control valves. The electrochemical reaction occurred in the main tank. The area of the cathode and anode in the main tank was 2 cm × 4 cm, and the interval between the electrodes was 2.5 cm. The auxiliary tank was directly connected to the temperature control device to keep the temperature of the electrolyte constant at 50 °C. The filter removed insoluble impurities, preventing them from impacting the experimental results during electrodeposition. The magnetic pump provided sufficient power for the electrolyte circulation system, and the flow rate of the electrolyte in the pipeline was controlled by adjusting the flow control valves. To perform electrodeposition of Ni single-layer coatings with a thickness of 50 μm, four different pulse current waveforms (Rec, Tri, Rup, and Rdn) were set by the pulse power supply, and the electrical parameters and electrodeposition time were carefully controlled for each waveform. These electrical parameters are summarized in Table 1. At T = 10 ms and θ = 0.5, two pulse combinations (Layer 1 and Layer 2) were used to electrodeposit multilayer coatings. The pulse current waveforms and pulse combinations diagrams are shown in Figure 2. Where T_on_ is the pulse on time, T_off_ is the pulse off time, and i_p_ is the peak current density. The thickness of Ni coatings and pulse electrical parameters of the Ni coatings were calculated by the flowing formula [23,24]:

(1) $$d=\frac{{\mathrm{Ki}}_{av}t\eta }{\rho }\times 10^{3}$$

(2) $$\eta =\frac{m}{\mathrm{ItK}}\times 100\%$$

(3) $$T=T_{\mathrm{on}}+T_{\mathrm{off}}$$

(4) $$\theta =\frac{T_{\mathrm{on}}}{T_{\mathrm{on}}+T_{\mathrm{off}}}$$

where d, K, t, η, ρ, m, and I are the coating thicknesses (μm), electrochemical equivalent (K_Ni_ = 1.905 g/A·h), plating time (h), density (ρ_Ni_ = 8.902 g/cm^3^), current efficiency, the mass of the deposit (g), and current intensity (A).

The high-purity Ni plate (99.99%) was employed as the anodic, while 304 stainless steel was used as the cathodic substrate. Before the experiment, the surface of the cathodic substrate was carefully polished using waterproof abrasive paper. Additionally, 10 vol % NaOH was used for alkaline cleaning for 5 min to remove organic pollutants in the cathode, followed by 10 vol % HCl pickling. Finally, the cathodic substrate was rinsed with deionized water to ensure a clean surface. The electrolyte was composed of 350 g/L Ni(II) tetrahydrate (Ni(SO_3_NH_2_)·4H_2_O), 10 g/L Ni chloride hexahydrate (NiCl_2_·6H_2_O), and 30 g/L boric acid (H_3_BO_3_).

### 2.2. Characterization

The surface morphology of the electrodeposited Ni coatings was observed by optical microscope (Olympus, OLS5100, Tokyo, Japan). To explore the layered structure of multilayer coatings, the cross-sectional morphology of the coatings was characterized by field emission scanning electron microscopy (SEM, Merlin Compact, Oberkochen, Germany). The grain orientation and crystallite size of the coatings were determined by X-ray diffraction (XRD, Smart Lab, Kanagawa, Japan). The relative texture coefficient (RCT) of each crystal plane of Ni coatings can be calculated using the formula [24]:

(5) $${\mathrm{RCT}}_{\left(\mathrm{hkl}\right)}=\frac{I_{\left(\mathrm{hkl}\right)}/I_{0(\mathrm{hkl})}}{{\Sigma (I}_{\left(\mathrm{hkl}\right)}/I_{0(\mathrm{hkl})})}\times 100$$

where I_(hkl)_ is the intensity of the diffraction peak from the Ni coatings, and I_0(hkl)_ is the standard sample from JCPDS data.

### 2.3. Corrosion Resistance

The electrochemical test was carried out in a standard three-electrode system, with a platinum plate (Pt) as the auxiliary electrode, a saturated calomel electrode (SCE) as the reference electrode, and the coatings as the working electrode. The corrosion resistance of the coatings deposited by the four pulse current waveforms and pulse combinations was evaluated through dynamic polarization curves in a 3.5 wt.% NaCl solution using an electrochemical workstation (CHI604E, CH Instruments, Shanghai, China). The coatings were polished with waterproof abrasive paper to remove the surface passivation layer before the test. Both the auxiliary and working electrodes measured 1 cm × 1 cm, and the scan was conducted at a speed of 5 mV/s within a potential range of −1.5 V to 1.5 V. The corrosion potential (E_corr_) and corrosion current density (I_corr_) were obtained by using the Tafer curve extrapolation method and the analysis software of the electrochemical workstation.

## 3. Results

### 3.1. Corrosion Resistance Analysis of Single-Layer Ni Coatings

#### 3.1.1. The Effect of Pulse Period

Pulse electrodeposition can effectively reduce the thickness of the diffusion layer, affect the nucleation rate of the crystal, and modify the corrosion resistance of the coatings by adjusting the pulse period [25,26]. To analyze the effect of the pulse period on the corrosion resistance of single-layer Ni coatings, electrodeposition was performed at an average current density (i_av_) of 40 mA/cm^2^ and a duty cycle (θ) of 0.5 using four pulse periods (T = 1, 10, 100, and 1000 ms) with four pulse current waveforms (Rec, Tri, Rdn, and Rup).

**Figure 3:**
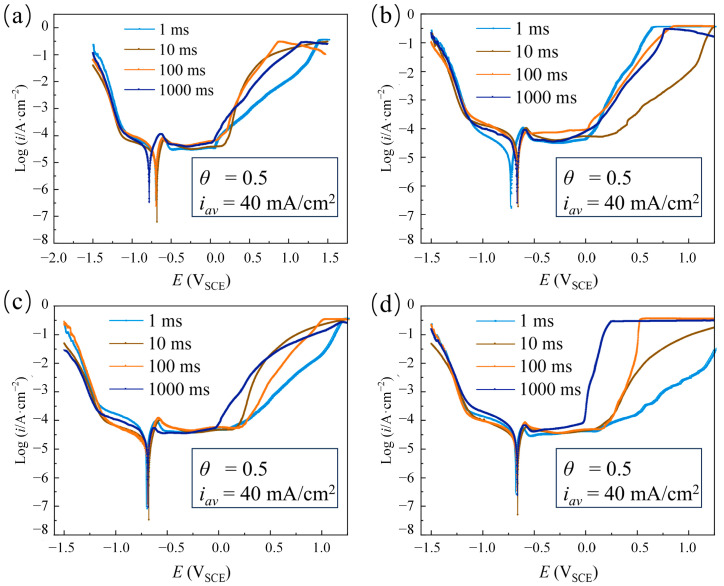
Dynamic polarization curves of electrodeposited Ni coatings at different pulse periods (T = 1 ms, 10 ms, 100 ms, and 1000 ms) and constant electrical parameters (θ = 0.5 and i_av_ = 40 mA/cm^2^) with different waveforms: (**a**) Rec; (**b**) Tri; (**c**) Rdn; and (**d**) Rup.

**Figure 4:**
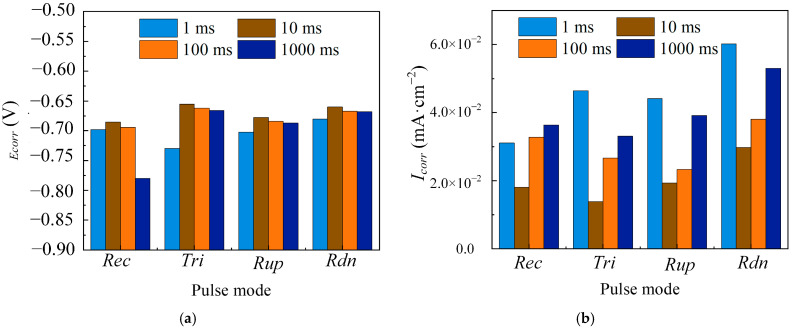
Different waveforms (Rec, Tri, Rup, and Rdn) electrodeposited Ni coatings at different pulse periods (T = 1 ms, 10 ms, 100 ms, and 1000 ms) and constant electrical parameters (θ = 0.5 and i_av_ = 40 mA/cm^2^). (**a**) E_corr_; (**b**) I_corr_.

**Figure 5:**
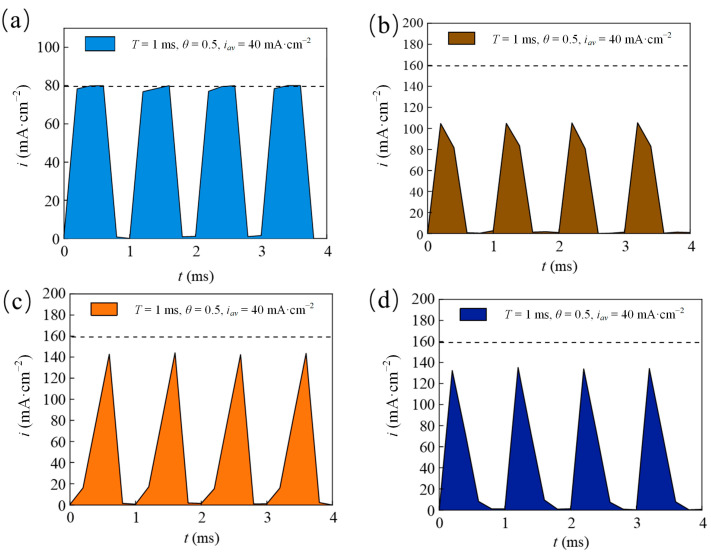
Pulse current waveforms detected at the electrical parameters of T = 1 ms, θ = 0.5, and i_av_ = 40 mA/cm^2^ with different waveforms: (**a**) Rec; (**b**) Tri; (**c**) Rdn; and (**d**) Rup.

Figure 3 shows that Ni coatings electrodeposited with the four distinct pulse current waveforms exhibited a clear active–passive transition behavior. As the pulse period increased, the pitting potential initially shifted positively and then negatively, while the passivation region initially expanded and then contracted. At T = 10 ms, the coatings electrodeposited with these waveforms exhibited a positive pitting potential and a broad passivation region. A quantitative analysis of the corrosion resistance was performed by examining the corrosion parameters under the four pulse current waveforms, presented in Figure 4. Compared with T = 10 ms, at T = 1 ms, the coatings electrodeposited with four different pulse waveforms showed lower E_corr_ and higher I_corr_. This can be understood as the distortion of the pulse waveforms in an ultrashort pulse period to the electric double-layer effect. Figure 5 shows that during the ultrashort pulse time, the electric double layer prevented the peak current of various waveforms from reaching the expected value, and the output waveforms were different from the input waveforms. Therefore, crystal growth might be incomplete, and pore flaws might emerge, leading to poor corrosion resistance [25]. As the pulse period increased to 10 ms and 100 ms, the E_corr_ of the Ni coatings deposited with the four pulse waveforms showed an upward trend, while I_corr_ decreased. It was shown that the current consumed by the double layer was small, and the induced current was large, resulting in a higher cathodic deposition overpotential. The nucleation rate of the crystal exceeded the grain growth rate, making the coatings more compact and enhancing corrosion resistance [27]. However, with an extremely large pulse period, the electroactive material in the cathode surface diffusion layer decreased faster than it could be replenished by the main solution. This led to a lack of Ni ions on the cathode surface, reducing the nucleation rate. Crystal growth then dominated electrodeposition, reducing the deposition layer sediment density and resulting in poor corrosion resistance [28].

In summary, the corrosion resistance of Ni coatings electrodeposited with the four pulse current waveforms initially increased and then decreased with the pulse period. The coatings demonstrated relatively good corrosion resistances at T = 10 ms and T = 100 ms. According to the theory of electrochemical deposition, a shorter pulse period corresponds to a thinner diffusion layer and a broader permissible range for the average current density [29]. Considering these factors, T = 10 ms was identified as the optimal parameter for further investigation of the effects of the duty cycle and average current density on corrosion resistance under the four pulse current waveforms.

#### 3.1.2. The Effect of Duty Cycle

The duty cycle in pulse electroplating is a critical parameter that significantly impacts the outcomes of the pulse electrochemical deposition process. When the current is turned off, hydrogen evolution bubbles are washed away by water flow, reducing internal defects in the coatings and effectively improving corrosion resistance [30]. To analyze the effect of the duty cycle on the corrosion resistance of single-layer Ni coatings, the coatings were electrodeposited at T = 10 ms and i_av_ = 40 mA/cm^2^, with different duty cycles (θ = 0.1, 0.3, 0.5, and 0.7) and four pulse current waveforms (Rec, Tri, Rdn, and Rup).

**Figure 6:**
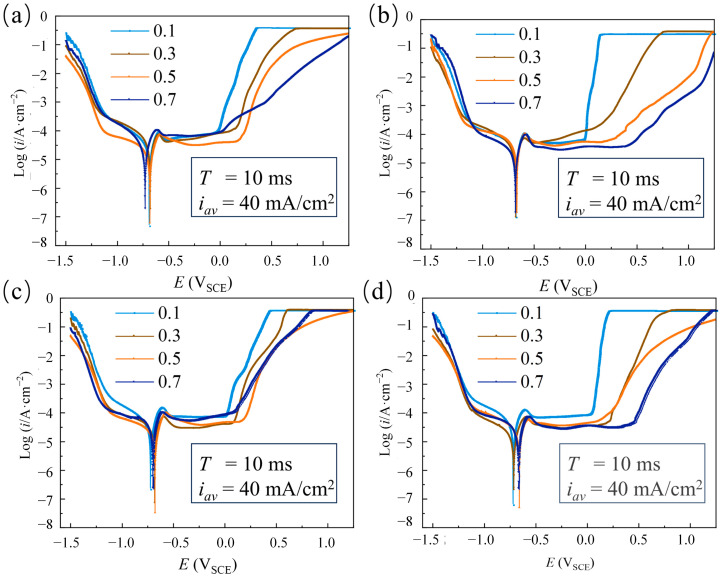
Dynamic polarization curves of electrodeposited Ni coatings at different duty cycles (θ = 0.1, 0.3, 0.5, and 0.7) and constant electrical parameters (T = 10 ms and i_av_ = 40 mA/cm^2^) with different waveforms: (**a**) Rec, (**b**) Tri, (**c**) Rdn, and (**d**) Rup.

**Figure 7:**
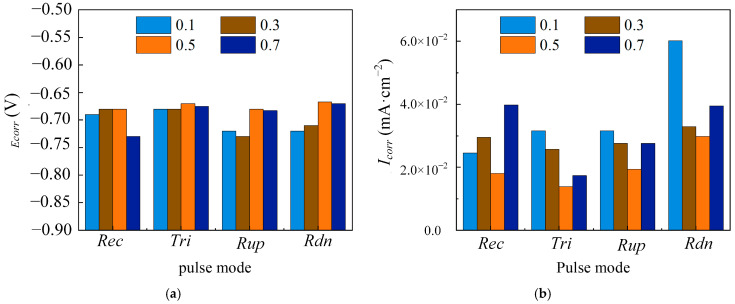
Different waveforms (Rec, Tri, Rup, and Rdn) electrodeposited Ni coatings at various duty cycles (θ = 0.1, 0.3, 0.5, and 0.7) and constant electrical parameters (T = 10 ms and i_av_ = 40 mA/cm^2^). (**a**) E_corr_; (**b**) I_corr_.

Figure 6 shows the dynamic polarization curves of the Ni coatings electrodeposited at different duty cycles with four pulse current waveforms. At a duty cycle of θ = 0.1, the pitting potential and passivation region for various pulse current waveforms were lower and narrower compared to those at other duty cycles. The coatings electrodeposited at θ = 0.5 with Rec and Rup, as well as coatings electrodeposited at θ = 0.7 with Tri and Rdn, exhibited relatively high pitting potentials and broad passivation regions. To further quantitatively analyze the corrosion resistance of the coatings with four pulse current waveforms, the corrosion parameters are presented in Figure 7. Compared with θ = 0.5, the coatings electrodeposited with four pulse current waveforms at θ = 0.1 had a lower E_corr_ and a higher I_corr_. It is believed that a large peak current density has a large cathode overpotential, resulting in serious hydrogen evolution on the cathode surface, loose and porous coatings, and poor corrosion resistance. The peak current density of Rec was 400 mA/cm^2^, while the peak current density of Tri, Rup, and Rdn was 800 mA/cm^2^. With the duty cycle increasing from 0.3 to 0.7, the E_corr_ of the coatings electrodeposited with four pulse current waveforms first increased and then decreased, and the I_corr_ first decreased and then increased.

**Figure 8:**
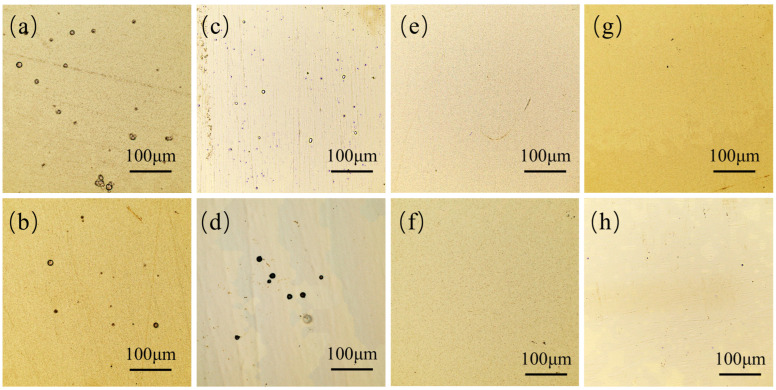
Optical microscopy images of electrodeposited Ni coatings with different waveforms and duty cycles at constant electrical parameters (T = 10 ms and i_av_ = 40 mA/cm^2^). (**a**) Rec, θ = 0.1; (**b**) Tri, θ = 0.1; (**c**) Rup, θ = 0.1; (**d**) Rdn, θ = 0.1; (**e**) Rec, θ = 0.5; (**f**) Rup, θ = 0.5; (**g**) Tri, θ = 0.7; and (**h**) Rdn, θ = 0.7.

This phenomenon was attributed to the combined effects of electrochemical and concentration polarizations. The large duty cycle can reduce the charging time of the electric double layer and increase the thickness of the pulse diffusion layer, resulting in an increase in the Faraday current and an increase in concentration polarization, thereby gaining a higher cathode overpotential [31]. However, when the duty cycle was large enough, the concentration polarization was considered large, and a large part was consumed in the hydrogen ion reduction reaction, resulting in a decrease in deposition efficiency, grain coarsening, and corrosion resistance. At the same duty cycle, the concentration overpotential decreased with Rup, Rec, Tri, and Rdn at the end of the T_on_ [32]. It is believed that Tri and Rdn reached the duty cycle of relatively good corrosion resistance of the coatings earlier than Rec and Rup. Figure 8 illustrates the surface morphology of the coatings formed by the four distinct pulse current waveforms at θ = 0.1, Rec and Rup at θ = 0.5, and Tri and Rdn at θ = 0.7. The results of morphological observation are consistent with those of dynamic polarization curve measurements.

Summarized, the corrosion resistance of the coatings electrodeposited at θ = 0.1 with four kinds of waveforms was poor. With the increase in the duty, the corrosion resistance of the coatings was enhanced to a certain extent. Specifically, the Rec and Rup waveforms demonstrated relatively good corrosion resistance at θ = 0.5, while the Tri and Rdn waveforms exhibited relatively good corrosion resistance at θ = 0.7. To prevent a large duty cycle from weakening the advantages of pulse electrodeposition, and considering that coatings electrodeposited with the four pulse current waveforms also showed relatively good corrosion resistance at θ = 0.5, subsequent research will select θ = 0.5 to analyze the effect of the average current density on the corrosion resistance of the coatings.

#### 3.1.3. The Effect of Average Current Density

The average current density is an important parameter in the pulse electrodeposition, controlling the nucleation rate and deposition rate of crystal [25]. To analyze the effect of average current density on the corrosion resistance of single-layer Ni coatings, the coatings were electrodeposited at T = 10 ms, θ = 0.5, and different average current densities (i_av_ = 10 mA/cm^2^, 20 mA/cm^2^, 40 mA/cm^2^, and 60 mA/cm^2^) with four pulse current waveforms (Rec, Tri, Rdn, and Rup).

**Figure 9:**
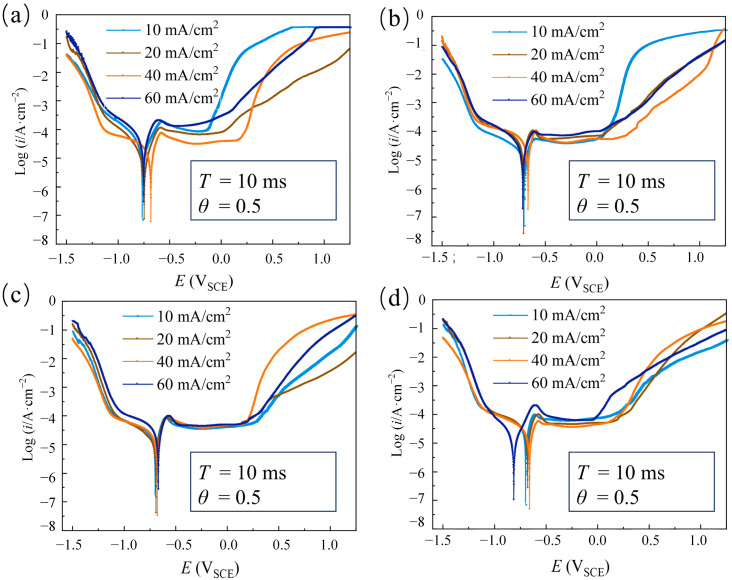
Dynamic polarization curves of electrodeposited Ni coatings at different average current densities (i_av_ = 10 mA/cm^2^, 20 mA/cm^2^, 40 mA/cm^2^, and 60 mA/cm^2^) and constant electrical parameters (T = 10 ms and θ = 0.5) with different waveforms: (**a**) Rec; (**b**) Tri; (**c**) Rdn; and (**d**) Rup.

**Figure 10:**
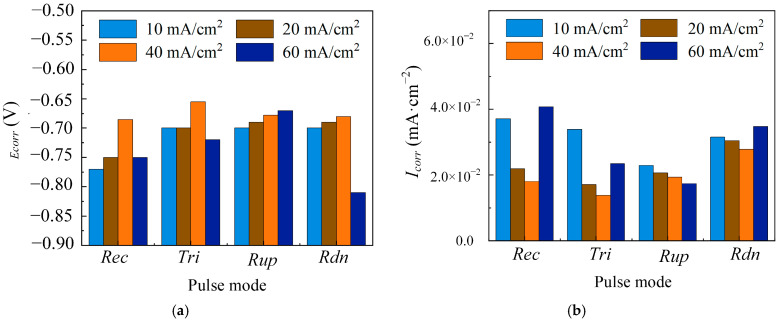
Different waveforms (Rec, Tri, Rup, and Rdn) electrodeposited Ni coatings at average current densities (i_av_ = 10 mA/cm^2^, 20 mA/cm^2^, 40 mA/cm^2^, and 60 mA/cm^2^) and constant electrical parameters (T = 10 ms and θ = 0.5). (**a**) E_corr_; (**b**) I_corr_.

Figure 9 shows that the pitting potential and passivation region of Ni coatings electrodeposited with Rec, Tri, and Rdn first increased and then decreased when the average current density increased from 10 mA/cm^2^ to 60 mA/cm^2^. However, for the coatings electrodeposited with Rup, the pitting potential and passivation zones continued to increase with a rising current density. To further quantitatively analyze the corrosion resistance of the coatings with four pulse current waveforms, the corrosion parameters are shown in Figure 10. The E_corr_ of the coatings showed an overall upward trend, while the I_corr_ decreased when the average current density increased from 10 mA/cm^2^ to 40 mA/cm^2^. The change in corrosion parameters can be understood as the average current density increasing the deposition overpotential, leading to higher crystal nucleation rates and finer grains [32]. The smaller grain size had better corrosion resistance due to its higher grain boundary density, which accelerated the formation of the passivation film [33]. As the average current density continued to increase to 60 mA/cm^2^, it was found that the E_corr_ of Rec, Tri, and Rdn coatings began to decrease, and the I_corr_ increased. The increase in the average current density can increase the polarization overpotential, which promotes the precipitation of hydrogen and competes with the deposition process. When the hydrogen evolution is severe enough, the dispersed and porous structure of the electrodeposition will be obtained, resulting in a decrease in corrosion resistance. However, the electrodeposited coatings at 60 mA/cm^2^ with Rup showed increases in E_corr_ and I_corr_. This can be understood as the concentration polarization during the pulse period being smaller than in other waveforms, resulting in less hydrogen evolution and better corrosion resistance [34].

By considering all of the above features, when the average current density increased from 10 mA/cm^2^ to 60 mA/cm^2^, the corrosion resistance of the Ni coatings electrodeposited by the Rec, Tri, and Rdn waveforms first increased and then decreased. Within the parameters of this experiment, the corrosion resistance of the coatings electrodeposited by Rup continued to increase with the increase in current density.

### 3.2. Corrosion Resistance Analysis of Multilayer Ni Coatings

Multilayer coatings, compared to single-layer coatings, can reduce pore size and defects through preferential nucleation and growth at high-energy defect sites of the preceding layer [21]. It is reported that the gradient structure multilayer coatings electrodeposited with dynamic current density can enhance the strength and reduce the corrosion resistance [35]. Compared with other waveforms, the coatings electrodeposited with Rdn have the finest grain size when the electrical parameters are the same [36]. With constant electrical parameters (T = 10 ms and θ = 0.5) and an increase in average current density from 10 mA/cm^2^ to 40 mA/cm^2^, the coatings electrodeposited with Rdn demonstrated relatively good corrosion resistance, and the dynamic polarization curve exhibited small fluctuations (Figure 9). To improve the corrosion resistance of the coatings further, we adopted the gradient current density electrodeposition with Rdn and proposed two pulse combinations (Layer 1 and Layer 2).

**Figure 11:**
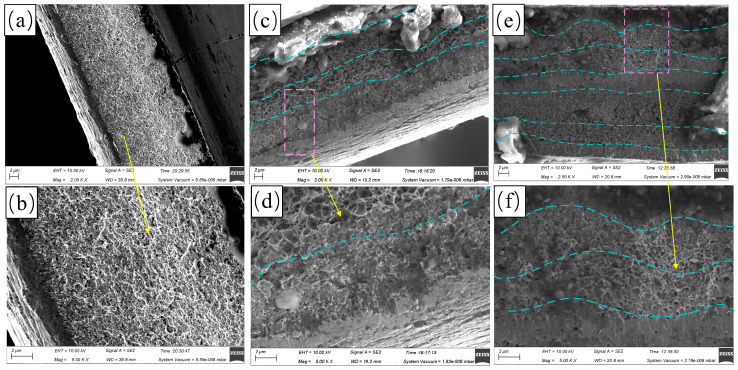
Cross-sectional morphologies of electrodeposited Ni coatings under different pulse combinations: (**a**,**b**) Monolayer; (**c**,**d**) Layer 1; and (**e**,**f**) Layer 2.

Figure 11 shows the single-layer Ni coating electrodeposited at electrical parameters (T = 10 ms, θ = 0.5, and i_av_ = 10 mA/cm^2^) with Rdn (Monolayer). These electrical parameters were the same as those of the outermost layer in the pulse combinations. The SEM images show the cross-sectional morphologies of the Ni coatings with different pulse combinations. The single-layer coating had a uniform, continuous structure with intersecting ravines. In contrast, the multilayer coatings displayed a pronounced stratified architecture. The intermediate layer of Layer 1 had a honeycomb structure, while the adjacent outer layer was more compact. The coating electrodeposited by Layer 2 exhibited similar characteristics, with honeycomb and dense layers alternately stacked to form a multilayer coating. Compared to the single-layer coating, the multilayer coatings fabricated using the combined electrodeposition technique were denser.

**Figure 12:**
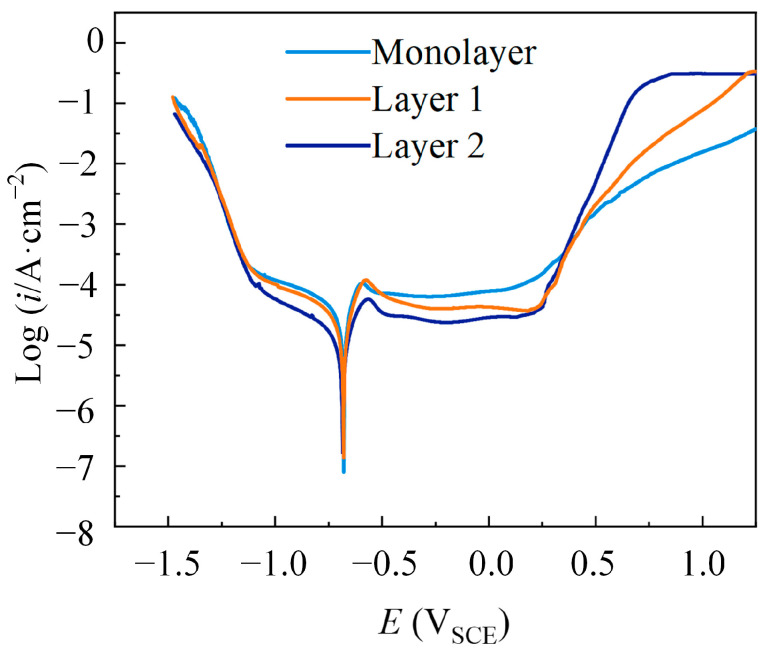
Dynamic polarization curves of coatings electrodeposited by Monolayer, Layer 1, and Layer 2.

**Figure 13:**
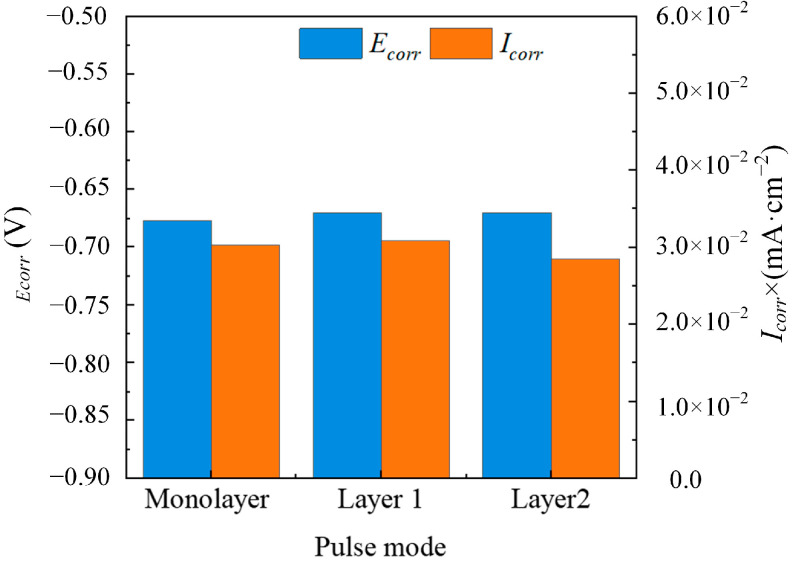
E_corr_ and I_corr_ of electrodeposition parameters with Monolayer, Layer 1, and Layer 2.

Figure 12 shows the dynamic polarization curves of Monolayer, Layer 1, and Layer 2. The pulse combinations had a slightly higher pitting potential than the single waveform, and Layer 2 showed a low passivation current density. The corrosion parameters obtained by the dynamic polarization curve are shown in Figure 13. The maximum differences in the electrical parameters with three coatings were similar (E_corr_ = 0.68 ± 0.01 V, I_corr_ = 3 × 10^-2^ mA/cm^2^). This similarity can be understood as it being the same electrical parameters of the outer layer of the coatings.

**Figure 14:**
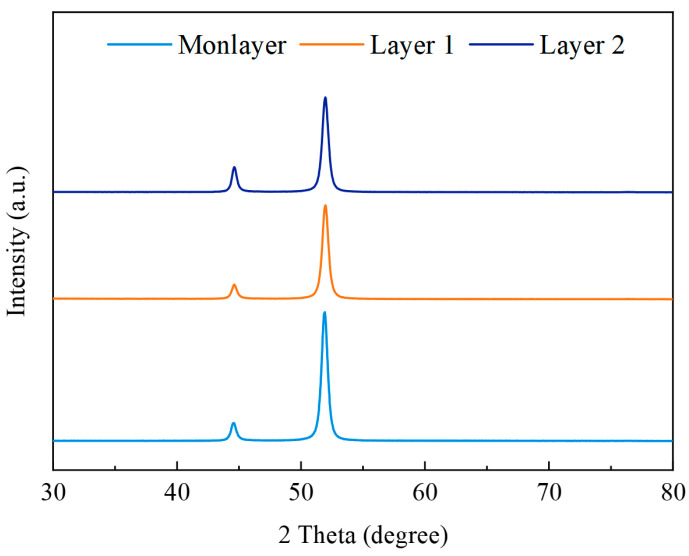
XRD patterns of the electrodeposits obtained with Monolayer, Layer 1, and Layer 2.

Figure 14 shows XRD patterns of the Ni coatings electrodeposited by Monolayer, Layer 1, and Layer 2. The coatings exhibited a face-centered cubic crystallographic structure with a pronounced (200) texture. The grain size was calculated by the Scherrer formula, and Monolayer was 50 nm, Layer 1 was 48 nm, and Layer 2 was 47 nm. The differences in grain size were minimal. Variations in passivation film performance within the passivation region may be due to changes in crystal structure. The (111) orientation had lower surface energy compared to the (200) orientation, making the passivation film form quickly and easily on the coatings with a (111) orientation. The relative texture coefficient (RCT) data are shown in Table 2. It was found that RCT_(111)_ decreased in turn with Layer 2, Layer 1, and Monolayer. This indicated that the (111) orientation of the coatings had higher corrosion resistance. This finding is consistent with the research of Yanqiu Yang et al. in 2020 [37]. This study showed that the Ni coatings electrodeposited with pulse combinations can improve corrosion resistance, and the coatings deposited by Layer 2 showed relatively better passive film and higher corrosion resistance.

## 4. Conclusions

Pulse electrodeposition effectively enhanced the corrosion resistance of Ni coatings. At the ultrashort and long pulse periods, coatings electrodeposited with four different pulse current waveforms showed a strong thermodynamic corrosion tendency or high corrosion rate. Within the parameters of this experiment, with the pulse period increasing, the corrosion resistance of coatings increased first and then decreased, reaching the best performance at T = 10 ms.

Adjusting the duty cycle can help reduce concentration polarization effects in Ni coatings during electrodeposition. The electrodeposited coatings at a low duty cycle exhibited relatively poor corrosion resistance and improved to a certain extent with increasing duty cycles. Within the parameters of this experiment, electrodeposited coatings with Rec and Rup performed relatively well at θ = 0.5, while Tri and Rdn performed relatively well at θ = 0.7.

The average current density also affected the corrosion resistance of Ni coatings produced by electrodeposition with pulse current waveforms. Within the parameters of this experiment, when the average current density increased, the corrosion resistance of electrodeposited coatings with Rec, Tri, and Rdn initially improved, reaching a peak at 40 mA/cm^2^, then declined. The corrosion resistance of electrodeposited coatings with Rup consistently improved with higher current densities.

Pulse combinations can further improve the corrosion resistance of electrodeposited Ni coatings. In this experiment, the passivation film of the Layer 2 coating demonstrated greater stability and higher corrosion resistance. Multiple layers had grain sizes ranging from 47 to 48 nm, with the highest RTC_(111)_ value observed in Layer 2, indicating a (111) grain orientation for improved corrosion resistance.

Future studies can focus on exploring the theory behind electrodeposited Ni coatings using pulse waveforms and investigating the corrosion resistance of multilayer Ni coatings electrodeposited by superimposing different pulse waveforms.

## Figures and Tables

**Table 1:** Electrical parameters of Ni single-layer coatings electrodeposition.

Parameters	Descriptions
Pulse current waveforms	Rec, Tri, Rup, Rdn
Pulse period T (ms)	1, 10, 100, 1000
Duty cycle θ	0.1, 0.3, 0.5, 0.7
Average current density i_av_ (mA/cm^2^)	10, 20, 40, 60

**Table 2:** Relative texture coefficients (RTC) electrodeposited with Monolayer, Layer 1, and Layer 2.

Pulse Combination	RTC_(111)_	RTC_(200)_
Monolayer	5.1	95.9
Layer 1	6.1	94.8
Layer 2	10.3	89.7

## Data Availability

Data are contained within the article.
